# Genomics workforce views on automating genomic reanalysis: trust, equity and governance

**DOI:** 10.1007/s00439-026-02824-7

**Published:** 2026-03-05

**Authors:** Emily A. King, Fiona Lynch, Zornitza Stark, Danya F. Vears

**Affiliations:** 1 Murdoch Children’s Research Institute, Parkville, VIC 3052 Australia; 2 University Hospital Geelong, Barwon Health, Geelong, VIC 3220 Australia; 3 Melbourne Law School, The University of Melbourne, Parkville, VIC 3052 Australia; 4 Deakin University School of Medicine, Waurn Ponds, VIC 3216 Australia; 5 Department of Paediatrics, The University of Melbourne, Parkville, VIC 3052 Australia; 6 Victorian Clinical Genetics Services, Murdoch Children’s Research Institute, Parkville, VIC 3052 Australia

## Abstract

Automation of genomic data reanalysis can increase diagnostic rates in a timely and cost-effective manner. However, implementing automated reanalysis brings with it both ethical and practical challenges. We sought to explore how clinical and laboratory staff view these challenges as part of a broader study piloting automated genomic reanalysis. Focus groups were conducted with 21 genetics health professionals recruited from a previous survey and purposively sampled from clinical genetics diagnostics services and laboratories. Participants were supportive of automating reanalysis but discussed trust and accuracy, and emphasized the need for transparency, updated phenotypic information and human checking in an automated model. Legal versus moral obligation to return results when patients are unable to be recontacted was extensively discussed. Participants held concerns about a potential legal obligation to return results arising as reanalysis becomes routine and potential for impacts on workflow. A centralized system with national governance was proposed to ensure development and implementation of an equitable service and facilitate communication between patients and genetics services. However, this may be limited by existing infrastructure and the public’s perception of government services. In conclusion, participants in this study supported automated reanalysis, however issues regarding transparency, trust and accuracy will need to be systematically addressed to support implementation.

**Supplementary Information:**

The online version contains supplementary material available at 10.1007/s00439-026-02824-7.

## Introduction

Next generation sequencing (NGS) technologies, such as exome and genome sequencing, have led to the ability to interrogate the entire genome to identify the cause of genetic conditions. Although these sequencing modalities have increased diagnostic rates compared to traditional testing methods, less than 50% of patients receive a diagnosis from their initial test (Chung et al. 2023). Yet, novel gene discovery, use of functional studies, updated phenotypic information, published evidence regarding variant pathogenicity and improvements in bioinformatics pipelines means that we are continuously increasing our potential to identify the cause of previously undiagnosable cases. In fact, studies show that reanalysis of genomic data, which can include reannotation, reprioritization and reinterpretation of variants using updated pipelines, can increase diagnostic yield by 10% (Dai et al. 2022).

This increased yield has led to calls from professional bodies to consider routine reanalysis (Deignan et al. 2019, Carrieri et al. 2019). This is because, currently, reanalysis is performed in an ad-hoc manner (Vears et al. 2020). Systems often rely on either the patient to reinitiate contact with the genetics service with a new referral, or a clinician to prompt the laboratory to reanalyze the data based on a new clinical presentation or new findings reported in the literature (Vears et al. 2020). An audit of Australian diagnostic laboratories estimated that 17,000 patients who received testing between 2018 and 2021 would benefit from reanalysis (Best et al. 2024). Despite the evidence for potential benefit from routine reanalysis, resource requirements severely limit our ability to achieve this at scale (Deignan et al. 2019). If the current model persists, few patients are likely to benefit from any reanalysis.

Automation has been proposed as a potential solution to the resourcing issues that would be required to reanalyze the large number of unsolved cases (Deignan et al. 2019, Coghlan et al. 2023, Sarmady and Abou Tayoun 2018). Automation of genomic data reanalysis refers to the use of computational pipelines to systematically reinterrogate previously generated genomic sequencing data as new knowledge emerges. This may include automated updating of variant annotations, integration of revised gene-disease associations, phenotype-driven variant prioritization, and rule-based or machine learning-assisted filtering and ranking of variants. Automation may involve fully rule-based systems, AI-assisted approaches, or hybrid models incorporating expert oversight. Although initial studies have shown that automation can effectively reanalyze previously undiagnosed clinical cases in a cost-effective manner (Matalonga 2021, Mensah 2022, O’Brien 2022), automation in healthcare remains a contentious issue. Furthermore, despite the clear precedent for the use of automation in genomic reanalysis, there has been little exploration of potential ethical concerns relating to large scale genomic data reanalysis.

We sought to understand the views of the workforce, patients and public associated with the use of automation in reanalysis in the context of a broader study developing and piloting a model of automated reanalysis (Fehlberg et al. 2024, Welland et al. 2025). This paper describes the ethical issues identified by the genomics workforce.

## Methods

### Sampling and recruitment

We aimed to recruit professionals currently working in an Australian clinical genetics diagnostics service or clinical laboratory. This included trainee and fully qualified clinical geneticists, genetic counsellors, associate genetic counsellors, clinical scientists, bioinformaticians and genetic pathologists. Participants were purposively sampled from a previously administered survey (Best et al. 2024) where they expressed interest in participating in further research. Clinical and non-clinical staff were also identified by contacting group leads and heads of departments at clinical genetics diagnostics services and laboratories across Australia. Participant selection aimed to enrich heterogeneity across departments, professional roles, levels of seniority, specialties, and Australian states.

Participants were invited to participate via direct email. They were invited to sign an online consent form and provide their contact details, professional details (role and organization), and availabilities if they wished to participate.

### Data collection and analysis

At the start of each focus group, author DV presented participants with information about the scale of the problem in Australia, including the number of undiagnosed cases in Australia (Best et al. 2024), current challenges to reanalysis, and a proposal for an automated genomic reanalysis pipeline. The proposed pipeline would automatically reanalyze currently negative cases. Suspicious variants thought to be either pathogenic or likely pathogenic, relating to the original reason for referral, would be flagged for formal assessment by the laboratory, and results returned to the requesting clinician.

Focus groups explored current practices with genomic reanalysis, practical and ethical considerations of implementing large-scale reanalysis using automation, and perceived impact on patient care and experience. The focus group guide is included as Supplementary Material.

Focus groups were conducted and recorded via Zoom by DV with the assistance of author FL in August, 2022. Focus groups were transcribed using an external transcription service and checked for accuracy by author EK. Transcripts were analyzed by EK using inductive content analysis, the process of generating codes from the data instead of using a predetermined coding structure (Vears and Gillam 2022). A subset of transcripts was co-coded by DV and all coding was discussed by EK and DV to resolve differences in coding, ensure agreement in the coding schema, and promote rigor in data analysis. Analysis was managed in NVivo 14 (2013).

## Results

### Participant characteristics

Twenty-one members of the genomics workforce participated in one of six focus groups (range two to six per group). Participant characteristics are summarized in Table 1.

**Table 1:** Focus group participant characteristics

Primary position	*n* (%)
Clinical	11 (52%)
Clinical geneticist (consultant)	3 (14%)
Clinical geneticist (trainee)	3 (14%)
Genetic counsellor	4 (19%)
Clinical immunologist	1 (5%)
Laboratory	10 (48%)
Clinical scientist	6 (29%)
Medical scientist	2 (10%)
Genetic pathologist	2 (10%)
Years of practice	
< 5	9 (43%)
6–10	3 (14%)
11–15	4 (19%)
16–20	1 (5%)
21–25	2 (10%)
25+	2 (10%)
TOTAL	**21**

### Perspectives on automating genomic data reanalysis

Our analysis identified three categories relating to the ethical issues associated with automating genomic data reanalysis: (1) benefits of an automated reanalysis pipeline; (2) challenges to establishing and integrating an equitable reanalysis service and (3) management of challenges of automated reanalysis. Representative quotes are used to illustrate these categories.

Although the information presented related to automation, participants tended to link this to AI technologies and we believed these data was relevant to include in the results.

An ellipsis (…) reflects where a significant portion of speech has been removed, and square brackets represent where a word has been replaced for clarity or to protect participant anonymity. Quotes are deidentified and labelled based on participants’ primary position and focus group number (e.g., FG1 refers to focus group 1).

### Benefits of an automated reanalysis pipeline

Participants were positive about an automated reanalysis pipeline and acknowledged that although its implementation would increase the workload in the short term, the benefits justified the required effort (Table 2, quotes 1 and 2).

**Table 2:** Perceived benefits of integrating automated genomic reanalysis to workload and patients

	Illustrative quotes
Initial bottleneck but benefits justify the workload	Quote 1: “17,000 unsolved cases, I can’t get it out of my head. So I feel like the benefits outweigh our workload.” [Medical Scientist 2, FG6] Quote 2: “I understand that that’s a massive resourcing bottleneck…if you could wave a magic wand and make it happen, I see absolutely no reason why we would not do it… clinically it’s important that they have updates as they become available.” [Genetic Counsellor 4, FG6]
Benefits	
Reduces wait times	Quote 3: *“our waiting list are really long so even once [patients] get a referral… they will still be sitting on a waiting list for months before we even see them to initiate that reanalysis. So*,* I think in the beginning [automated reanalysis] would be great”* [Genetic Counsellor 3, FG4]
Improves turnaround times	Quote 4: *“So*,* if the way I see this reanalysis pipeline is that it bypasses the prioritisation step*,* I think it really cuts down a lot of time in our turnaround time.”* [Clinical Scientist 1, FG2]
Reduces burden on patients and their families	Quote 5: *“[patients reinitiating contact is] putting the onus back on people who are already dealing with lots of other things with their rare diseases… this automated trigger would be*,* in my mind*,* it would work”* [Genetic Counsellor 4, FG6]
Decreases those lost to follow up	Quote 6: *“we worry about how many people are getting lost because we just don’t have the time or capacity to do that follow up or people that could have a diagnosis and are sitting there waiting”* [Genetic Counsellor 3, FG4]
Reduces workload over time	Quote 7: *“Once the technology becomes the norm*,* then we won’t be seeing people as much in follow-up… less of those follow up review appointments. But that’ll take a couple of years”* [Genetic Counsellor 2, FG3]

Participants believed it would improve the patient experience by reducing genetics service wait times for reanalysis (Table 2, quote 3), turnaround times (Table 2, quote 4), patient-initiated contact (Table 2, quote 5), and those lost to follow up (Table 2, quote 6). Clinical and laboratory staff also anticipated that an automated pipeline could reduce their workload by streamlining steps in reanalysis and reducing the number of review appointments (Table 2, quote 7).

### Challenges to establishing and integrating equitable reanalysis

#### Impact of automated reanalysis on service delivery

Participants highlighted that the ability of their service to reanalyze data would be limited by resourcing issues and associated costs (Table 3, quote 1). There were concerns that reanalysis cases would increase current, already lengthy, genetics services wait times (Table 3, quote 2). Participants noted that Australia’s healthcare system requires patients to obtain a new referral to see genetics services after a period of time (Table 3, quote 3) which would add further burden on reanalysis patients who are already under incredible amounts of strain.

**Table 3:** Participant-identified challenges to an automated reanalysis pipeline

	Illustrative quotes
Challenges relating to service delivery	
Resource limitations	Quote 1: *“It mainly comes down to costs and resources of course… genetic pathologists are extremely rare and in high demand at the moment.”* [Clinical Scientist 4, FG5]
Compound existing genetic services wait times	Quote 2: *I just think it’s going to be really difficult to manage amongst other priorities… how do you see everyone who is equally urgent within a reasonable timeframe without extra workforce really.”* [Genetic Counsellor 3, FG4]
Need for a new referral	Quote 3: *“If they want a reanalysis… they would need to be rereferred*,* they would need to have a new discussion… Some patients feel that that’s again an added step*,* something else they have to do.”* [Clinical Geneticist 5, FG1]
Risk of false positives	Quote 4: *“Are we going to get more false positives? Because if we do*,* then that can actually have harmful effects for patients.”* [Genetic Pathologist 2, FG5]
Need for updated phenotypic information	Quote 5: *“They can’t expect us to find these new associations if we’re not given the complete clinical picture.”* [Clinical scientist 5, FG1]
Need for clinician data entry	Quote 6: *“I’ve worked with clinicians who absolutely refuse to enter any kind of data entry. No matter how small*,* they will fill it out on paper*,* they will send an email*,* they will do that but they will not actually log in and actually tick a box.”* [Genetic counsellor 4, FG6]
Inappropriate counselling by non-genetics workforce	Quote 7: *“[non-genetics professionals] might feed it back to the family*,* but not really understand the wider implications”* [Genetic Counsellor 2, FG3]
Challenges relating to technology	
Appropriateness of sequencing technology used to generate genomic	Quote 8: *“Are you limiting it only to cases which are based on [an] exome backbone…it’s higher yield in terms of reanalysis versus just like a mendeliome or Invitae*,* gene X panel.* [Immunologist, FG6]
Transparency in the artificial intelligence pipeline	Quote 9: *“I still would want to know what happened before that*,* it gives me a better understanding of what I’m dealing with… to understand the limitations of what comes out of it.”* [Clinical Scientist 3, FG4] Quote 10: *“Unless a lot of really weird and unfortunate variants keep falling out that I might question it because it’s like*,* well*,* none of these are really panning out*,* do we need to review the process. So that they’re not yielding*,* but presuming that everything’s fine*,* that it’s doing its job*,* I’m not really…yeah*,* I don’t really understand it all that well so it’s going to be wasted on me to understand the process”* [Clinical Scientist 5, FG6]
Need for human input	Quote 11: *“…automatic reanalysis that’s machine based still requires human review because we need to make sure that it’s real and that it has a real impact for the patient or patient management”* [Genetic Pathologist 2, FG5]
Challenges relating to governance, moral duty and liability	
Who should oversee implementation	Quote 12: *“Everyone uses different software at the moment so if one laboratory is using one piece of software for the primary analysis and then the automated reanalysis is using something else then the original results may differ*,* so it may have to be the original laboratory reanalysing through their original pipeline.”* [Clinical Scientist 4, FG5]
Legal obligation to return results when unable to recontact the patient	Quote 13: *“How do you contact the patient? They may have moved*,* and then the issue of if you have a result*,* what’s the legal obligation of disclosing that result if you can’t get hold of that patient?”* [Clinical Geneticist 1, FG1]
Boundaries of moral responsibility to disclose results	Quote 14: *“what if a variant was flagged for us to curate it and then we receive notification that the family has withdrawn consent and then we’re like*,* but we’ve seen this variant now*,* we can’t unsee it.”* [Clinical scientist 1, FG2]
Risk to clinical relationship	Quote 15: *“it doesn’t take very much for us to lose a great deal of trust in the public and actually cause a great deal of reputational and financial damage if we don’t do it in the proper way from the get go.”* [Genetic Pathologist 2, FG5]
When are diagnostic results considered part of the medical record and how will this impact insurance	Quote 16: “*my questioning is about insurance: at what point*,* because it is a diagnostic test and a diagnostic result once you’ve found something*,* at what point is that information considered part of their medical record or not?”* [Genetic Counsellor 3, FG4]

Participants also raised concerns regarding the accuracy of results generated by an automated reanalysis pipeline and the risks of false positives (Table 3, quote 4). Participants stressed the need to routinely update clinical information to ensure an accurate result from reanalysis (Table 3, quote 5) however, one genetic counsellor noted that quality of clinician data entry may impact the success of the pipeline (Table 3, quote 6). Participants were also concerned that patients may be inappropriately counselled by non-genetics health professionals returning results (Table 3, quote 7). Participants raised concerns about how these factors may impact the equitable implementation of a reanalysis service and exacerbate existing inequities.

#### Concerns regarding accuracy of automation in reanalysis

Further to workflow issues, participants questioned the appropriateness of the type of sequencing technology used to generate the initial genomic data (Table 3, quote 8) in terms of accuracy and diagnostic yield, and therefore whether its quality would be appropriate for reanalysis.

Additionally, participants’ perspectives varied on whether they would need to understand the automated reanalysis pipeline to feel comfortable with its use. Some needed transparency in the pipeline to understand its limitations and to have the ability to review decisions made during reanalysis (Table 3, quote 9). However, other participants explained they thought it was only important to understand the pipeline if there were concerns about the results being generated (Table 3, quote 10).

Although clinicians and laboratory staff supported an automated process, they still thought it was essential to have a final check or review performed by a person before disclosure of the result (Table 3, quote 11).

#### Governance, moral duty, and liability

Participants were unsure about who would be best suited to oversee implementation of automated reanalysis. One issue raised was that laboratories use different software so implementation may need to be overseen at a local level (Table 3, quote 12).

When discussing recontacting patients to return results, participants raised concerns regarding legal and moral duties and potential implications. Some were concerned about their obligation to return a positive result if they were unable to locate and contact the patient. Others were concerned about the legal implications of not returning a positive result to a patient who withdraws consent after a positive result has been identified (Table 3, quote 13). Participants described a moral duty to return results in both instances and were particularly conflicted about situations where patients may have declined reanalysis or receiving results (Table 3, quote 14). There were concerns that if consent and result disclosure were not handled appropriately, then there would be significant risk to the reputation of and trust in the healthcare service (Table 3, quote 15).

One genetic counsellor queried when a positive result would be uploaded into the medical record and how this may impact a patient’s insurance (Table 3, quote 16).

#### Management of challenges of automated reanalysis

Participants debated how reanalysis cases should be prioritized against existing cases. Views ranged from prioritizing reanalysis cases for an ‘easy win’ (Table 4, quote 1), to deprioritizing them completely (Table 4, quote 2). Others thought reanalysis cases should be triaged using existing processes (Table 4, quote 3) and it should be adjustable to incorporate changes in clinical urgency, such as reproductive planning, pregnancy or patient deterioration (Table 4, quote 4).

**Table 4:** Proposed strategies to address service delivery and technological challenges to implementation of an automated reanalysis pipeline

	Illustrative quotes
Potential triaging methods for reanalysis cases	
Reanalysis cases prioritized over existing cases	Quote 1: *“they would just go to the top of the pile*,* which means that the bottom pile just keeps getting bigger… Because deep down we like to give a result back*,* because all the times that we don’t get a result*,* it’s nice to actually get one for a change.”* [Genetic Counsellor 1, FG3]
Prioritize existing cases	Quote 2: *“if the reanalysis wasn’t a thing*,* then the patient wouldn’t have been reanalysed. So …it has to be at the lowest round of priority*,* right? Because all the other patients actually have someone clinically managing them*,* but the ones that are being flagged for reanalysis don’t necessarily have an active clinical episode”* [Genetic Pathologist 1, FG2]
Reanalysis cases triaged alongside existing cases using the same process	Quote 3: *“I think we would*,* that would just go into the standard triaging mix and consideration about how urgent that was and how that patient can be seen. So*,* that will probably mean an update of clinical prioritisation criteria around triaging to accommodate these patients.”* [Clinical Geneticist 4, FG1]
Prioritize based on clinical urgency in response to updated clinical information	Quote 4: *“Because if there’s no clinical urgency*,* yeah*,* you’ll just be at the bottom of our list unless they say*,* oh*,* the child is now critically ill*,* or the parents are planning for a second baby.”* [Clinical scientist 1, FG2]
Centralized system with national oversight	
Implementation should be facilitated by a centralized system	Quote 5: *“if we had a national electronic medical record system that the software could access*,* you wouldn’t then rely on someone feeding us new information. You’d pull phenotype terms from an electronic medical record system?”* [Clinical Geneticist 1, FG1] Quote 6: *“Yes*,* maybe like a centralized platform and maybe if we talk about the new reports coming out*,* maybe we can add it to a portal and once it gets uploaded to our patient*,* the clinician just gets an email notification or something and then hopefully they will just check the portal.”* [Medical Scientist 2, FG6] Quote 7: *“They’ve moved interstate and you’ve got jurisdictional issues*,* we can’t see patients interstate*,* but I’m the ordering clinician for that so there are some logistical things”* [Clinical Geneticist 4, FG1]
Oversight from national body	Quote 8: *“I’m very sure many labs would not want to give up control over their data but I think if you’re going to have the one automated pipeline to rule them all and be useful*,* then it might be better to have this data repository for it and that it’s done at a national level with appropriate governance and oversight and experts involved in that.”* [Genetic pathologist 2, FG5]
Cost considerations	Quote 9: *“If you say it’s a thousand dollars a year or something*,* yes*,* people will be disadvantaged*,* but if you made it…I don’t even know what reasonable is… But it also highlights the value of the expertise*,* right? It doesn’t have to be extortionate in price but we can’t expect everything to be done for free.* [Genetic Counsellor 4, FG6]

To address both governance and technological issues, participants were in general agreement that implementation of automated genomic reanalysis would be best supported by a centralized system – a national online and uniform system that facilitates communication and input between patients, clinicians and the laboratory. This kind of system would allow clinicians to update phenotypic information (Table 4, quote 5), notify treating clinicians about upcoming reanalysis results (Table 4, quote 6), and address jurisdictional issues that arise when patients change healthcare services or move interstate (Table 4, quote 7).

To ensure efficiency and equity of a reanalysis pipeline, one participant suggested national governance would be necessary (Table 4, quote 8). Regarding funding, one participant suggested a fee-for-service model as a potential model for reanalysis (Table 4, quote 9), although there was contention amongst participants in relation to this proposition.

## Discussion

Our study explores the genomics workforce views on the use of automation in genomic reanalysis for undiagnosed cases and provides an in-depth analysis of associated ethical issues. Our findings demonstrate support from the genomics workforce for an automated reanalysis process. However participants raised concerns regarding potential legal and moral obligations for recontacting patients and return of positive results. Participants highlighted the need for transparency and a human checking mechanism to maintain trust in the pipeline. They suggested using a centralized system to update patient contact information and collate updated phenotypic data to incorporate into the algorithm.

### Facilitating trust in, and accuracy of, an automated pipeline

Our participants raised concerns regarding the quality of the genomic data to be used in reanalysis. They questioned how updated phenotypic information would be collected and incorporated into the pipeline if it became automated and did not require clinician re-referral. Increased diagnostic rates from reanalysis are, in part, due to updated phenotypic information guiding gene selection, and improvements in bioinformatics pipelines (Dai et al. 2022). Reanalysis requires the most up to date patient information to generate an accurate result. However, without a method to collect updated data at scale, reanalysis is unlikely to yield meaningful or accurate results. Should an AI system be incorporated in future, the accuracy of data would be even more important to train data sets.

Whilst our participants recognized the potential benefits automation would bring to genomics, they still expressed concerns given the potential for, and risk of, misdiagnoses. To ensure trust in automation, transparency in the pipeline and a final check performed by a human to validate the accuracy of a result was deemed essential. Similarly, transparency in healthcare AI, or lack thereof, has been a point of contention. Some have argued that AI cannot be adopted given the high-stakes nature of treatment decisions (Wadden 2022) and the risk that healthcare providers may uncritically accept results (Morley et al. 2020). Others counter that excessive distrust can prevent the beneficial adoption of AI (Coghlan 2023). Whilst some level of transparency is achievable in automation using AI technologies, it is impossible with ‘black box’ systems, such as deep learning approaches, where automated algorithms are not visible to those that created it (Coghlan 2023). The inevitable integration of AI adds another layer of complexity. However, any automation, including machine-based learning or deep learning AI systems, needs a degree of transparency to balance the concerns of the workforce, risk of misdiagnoses, and benefits of AI, to establish trust from the workforce.

### Workforce implications

Some of the key concerns of our participants related to the challenges they foresaw in service delivery if automated reanalysis was implemented. These included ensuring clinical information is up to date, a need for new referrals, and impacts on waiting times. Moving forward, we would be advocating for a model where unsolved cases are automatically included in automated reanalysis pipelines, avoiding the need for another referral. Although the workload at the time that automated reanalysis is launched will potentially increase, the iterative nature of the reanalysis means that, over time, diagnoses will resemble a trickle rather than a gushing faucet. Our findings highlight a need to increase awareness of the processes involved in automated reanalysis if it is going to be performed routinely, iteratively, and at scale.

### Perceived duties to return positive results

^3,43,4,1818^Our participants were uncertain about how far they should go to fulfill their perceived obligation to recontact a patient, especially given the complexities posed by the duty to warn. In Australia, there is no definitive advice regarding the extent to which one must attempt to follow up a result with a patient. For standard pathology and imaging, the Australian Medical Association suggests doctors “make a reasonable attempt to inform a patient who has a clinically significant result or diagnosis” (Australian Medical Association 2024). As time from their initial genomic test passes, patients are likely to have moved without updating their contact details with hospitals and healthcare providers may be left with what they perceive to be an impossibly large task of recontacting patients. In one study, a UK doctor spent five hours contacting hospitals in multiple countries to return a result for one patient (although this is an extreme example) (Mitchell et al. 2020). Recontacting patients will be limited by jurisdiction restrictions and may be perceived as an intrusion of privacy and breach of their right not to know (Carrieri et al. 2019). With resource limitations already challenging the integration of automated reanalysis, we need to consider how feasible it is to ask such extensive efforts of clinicians to recontact patients and more data is required to understand the actual workload implications.

This ethical obligation to return a positive result became even more concerning to participants in contexts where patients have not given consent to reanalysis. They queried whether positive results would be uploaded into the medical record in situations where the result had been returned to a patient. If this situation arose, it would raise the possibility of patients receiving reanalysis results from non-genetics health professionals. We suggest it is important to ensure that those who might be asked to disclose results have sufficient training to do so in a supportive way.

Our participants also held concerns about how automated reanalysis might have insurance implications for participants. This included if they had cancelled their insurance after the initial test, or if the patient could not be recontacted and their result was uploaded to their medical records without their knowledge and the insurance company penalized them for not disclosing their result. In reality, these are all very unlikely outcomes (and in Australia, at least, soon to be irrelevant as new laws are introduced to prevent insurance companies using genetic data in this way) (Tiller 2025). However, the fact that our participants were worried about these issues points to a need for specific training to improve clarity.

### Current and future challenges to provision of an equitable automated pipeline

Although our participants believed an automated pipeline would eventually improve wait times and reduce workloads, they also identified challenges to the equitable provision of services. Participants held disparate views on the best way to triage reanalysis cases, with some thinking they should be deprioritized completely and others thinking they should be returned first. The difficulty in reaching consensus is likely due to resource limitations (Carrieri et al. 2019) and potential concerns that prioritizing reanalysis cases over new cases creates inequity. Others have noted that timing of genomic testing without reanalysis is a predictor of poorer outcomes and leads to inequity (Hall and Raza 2014) as earlier patients did not receive the same standard of service that is available today. As one participant suggested, applying the existing triage process to reanalysis cases and incorporating changes to clinical urgency may be one way of facilitating an equitable service for both undiagnosed and new cases.

Interestingly, although participants identified the risk of false positives with automated reanalysis, they did not address how automation may perpetuate existing inequities in marginalized groups, previously identified as a major concern for the public (Harrison et al. 2024). An automated pipeline would be trained using current genomic data and identified diagnoses. However, studies have shown that VUS rates are higher in ethnic and racial minority populations, in part due to disparities in access to genetics services (Watts 2023), and over-representation of European ethnicity in population studies (Clarke and El 2022). Automation and AI tools that have been developed in healthcare have been shown to produce inaccurate results in different ethnicities, such as one tool in China that was trained on Western data, which produced poorer results for Chinese patients (Liu et al. 2018). Findings from focus groups with members of the public (Harrison 2024), in line with other research (Vokinger 2021), propose that development of an automated pipeline should implement de-biasing steps in its development to mitigate the risk of unjust bias and discrimination against individuals or groups. We did not collect demographic data about participants outside of primary position and years of practice. Further research that collects this information, such as ethnicity, is needed, as well as more specific exploration to see if the workforce is concerned about bias in an automated pipeline, or if other issues related to equity and transparency are more critical.

Despite the challenges, participants recognized that an automated pipeline could facilitate the equitable provision of reanalysis. Reanalysis is currently performed in an ad hoc manner; it can be initiated by the patient, clinician or laboratory, and varies between health services (Vears et al. 2020). As such, the system relies on particularly motivated patients to reinitiate contact and there is no way to ensure all patients who may benefit from reanalysis are identified or tested (Carrieri et al. 2019). In contrast, an automated pipeline would introduce a standardized approach to provision of reanalysis, ensuring access for all patients to reanalysis. Additionally, once the pipeline is implemented, the workforce will see a reduction in workload and turn-around times will improve, meaning more patients receive results in a timelier manner.

### Governance structures: local versus a centralized electronic system

Participants discussed local versus national oversight for the integration of automated reanalysis into practice. Although a local approach may be easier for individual laboratories given differences in systems, there is a risk of variable quality of the algorithm such that initial testing service is a determinate for quality of reanalysis results. Instead, a centralized system can facilitate communication with patients so they themselves can update contact details, phenotypic information, and family history, and request updates at life cycle junctions (David et al. 2019). Some countries have existing national health platforms that could incorporate automated reanalysis programs, which would streamline logistics and enable seamless integration into existing healthcare funding models (The The Commonwealth Fund 2024). 

However, studies have shown that the public is wary of both private and government entities overseeing the governance of genomic data, with concerns regarding security and misuse of data. Because of this, the public demonstrates strong preferences for strict regulation to protect data and its use (Lynch et al. 2023). As a result of these concerns, patients may choose to opt out to programs overseen by government, reducing the benefit of automated reanalysis. However, privately-owned companies also pose risks associated with sharing health information and for-profit use of genomic data. Alternatively – as suggested in focus groups with the Australian public exploring genomic data governance – data could be overseen by a purpose-built non-government organization or not-for profit organization (Lynch et al. 2023). However, this would require significant frontloading of resources and data transfer (Harrison et al. 2024). At this stage, although a centralized system promises to address many challenges associated with an automated reanalysis pipeline, lack of trust in government organizations could hinder progress in some countries.

This study was undertaken in an Australian context where there is a universal healthcare service. As such, considerations for automated reanalysis may not be applicable in all contexts. In line with this, we adopted a qualitative exploratory approach which aimed to generate insights, rather than produce generalizable findings. This was also part of a wider evaluation strategy, with results of audit and surveys of clinical and laboratory practice already published (Best et al. 2024) and a discrete choice experiment to follow. Together, these will inform the implementation of automation in genomic reanalysis in Australia and more globally.

This paper does not address all ethical issues associated with automated reanalysis. Whilst there was some discussion in the focus groups regarding cost and insurance implications of automated reanalysis, this was not discussed in detail. As such, it would be important to further explore these factors given the significant financial burden associated with establishing large-scale reanalysis and the difference in insurance policies globally.

Automation may reduce time, labor and costs; however, it poses new ethical considerations to genomic reanalysis. This study demonstrates that human oversight is crucial in establishing and maintaining trust from the workforce. Focus groups discussions showed that scaling reanalysis through automation brings to the fore issues about operationalizing patient recontact in an equitable manner, which need to be considered prior to implementation to minimize the potential for harm and moral distress. Further work is needed to understand the impact of automated reanalysis to healthcare systems globally, and to set up appropriate clinical, laboratory and funding models that optimize patient and service benefit.

## Supplementary Information

Below is the link to the electronic supplementary material.

Supplementary Material 1

## Data Availability

Redacted transcripts available on request.
