# Supplementary material for: Genomics workforce views on automating genomic reanalysis: trust, equity and governance
Source: Hum Genet. 2026 Mar 5;145(1):29. doi: 10.1007/s00439-026-02824-7 (PMC12963070; doi:10.1007/s00439-026-02824-7)
Supplement: Supplementary file 1 — Supplementary Material 1 [file 439_2026_2824_MOESM1_ESM.docx]

**Supplementary Material 1: Focus group interview guide**

Note: This document acts as a guide to the interviewer. Exact language and order of questions varied based on participant discussion.

**WELCOME**

Thank you for agreeing to be part of this 90-minute focus group discussion. We appreciate your willingness to participate.

My name is [interviewer’s name]. I’m a [interviewer’s position, academic degree, and affiliation], and with me today I have [second interviewer’s name], who is [second interviewer’s position, academic degree, and affiliation].

We’ll get you all to introduce yourselves shortly.

**PURPOSE OF THIS SESSION**

We are conducting research exploring the ethical, practical and economic issues of the automation of reanalysis of genomic sequencing data.

We want to hear your thoughts on a proposed model of reanalysis on behalf of those who will be impacted most by this approach.

There are a lot of things that need to be considered when making decisions about how automated reanalysis should be done, including:

- Whether and to what degree it will fit into your existing workflows
- Whether reanalysis should be routinised or triggered in some way
- What impact this will have on patient care

The purpose of this discussion today is to understand the aspects about these topics that are important to you. We want to know which aspects you think are good and also which ones you think may not be so positive. What risks and benefits do you identify with these different aspects of automated reanalysis?

**BACKGROUND**

[Delivered with PowerPoint presentation]

Reanalysis of genomic sequencing data is currently performed manually by laboratories on a case-by-case basis, with reports issued back to requesting clinicians.

Reanalysis is currently funded in an ad-hoc manner through clinical services from existing budgets.

Recently, Medicare funding became available for example for those children who have had Medicare-funded testing for syndromic and non-syndromic ID. Medicare will fund two cycles of reanalysis, at least 18 months apart, up to the age of 15. This item will be available from November 2022 onwards. Similar items are tied to newer genomic MBS item numbers (e.g. inherited kidney disease). These can be requested by clinical geneticists or appropriate specialist physicians (e.g. paediatricians).

We are proposing to automate the process of reanalysis. In this model, genomic data from unsolved cases will be re-aligned, re-annotated and continuously interrogated to identify likely pathogenic and pathogenic variants related to the original reason for testing. These variants may be identified through reanalysis, for example, as a result of newly published gene-disease associations or identification of new variant types, such as small deletions or short tandem repeats. This is expected to benefit thousands of patients that do not have a diagnosis.

***Key considerations***

- This automated reanalysis pipeline will be designed so that negative cases are automatically reanalysed in an iterative cycle without requiring clinician-initiated requests.
- Highly suspicious variants flagged by the automated reanalysis program will be formally assessed by diagnostic laboratories and pathogenic/likely pathogenic findings will be reported to the original requesting clinicians.

**INTRODUCTION**

Now it’s your turn to talk! First, when I call on you, I’d like you to introduce yourselves with your first name and your professional role so we can get to know each other.

**GROUP DISCUSSION**

Now we would like your thoughts on automated reanalysis of genomic sequencing data.

- How would the automated reanalysis model we presented fit into your current workflow?
  - How would automated reanalysis impact your current workflow?
    - In what ways would automated reanalysis improve your current workflow?
    - In what ways would automated reanalysis place additional burden on your current workflow?
  - What would need to change for it to fit? (either the model or the current work flow)
- What should the process of automated reanalysis look like?
  - Should reanalysis be ‘triggered’ or routinised? If triggered, by what/whom?
  - Who should results be returned to and how?
  - How will results be conveyed to patients? By whom?
  - How often should reanalysis be done/offered?
- What should consent for automated reanalysis look like?
  - Is it already covered by existing consent forms?
  - Who should be responsible for obtaining consent?
  - Should patients/health professionals get to opt out of reanalysis, and how?
- How should the reanalysis be funded?
- What sort of impact do you think automated reanalysis will have on patient care?
  - How do you perceive automated reanalysis improving patient care?
  - How do you perceive automated reanalysis negatively impacting patient care?
- How confident do you feel about being able to deliver automated reanalysis (both short term and long term) within your service?
- Do you have any other concerns about the introduction of automated reanalysis?

**WRAP UP**

- Request any final comments and thoughts from participants
- Summary of discussion
- Next steps
